# Prevalence of Epithelial Ovarian Cancer Stem Cells Correlates with Recurrence in Early-Stage Ovarian Cancer

**DOI:** 10.1155/2011/620523

**Published:** 2011-08-29

**Authors:** Karina Dahl Steffensen, Ayesha B. Alvero, Yang Yang, Marianne Waldstrøm, Pei Hui, Jennie C. Holmberg, Dan-Arin Silasi, Anders Jakobsen, Thomas Rutherford, Gil Mor

**Affiliations:** ^1^Department of Oncology, Vejle Hospital, Kabbeltoft 25, 7100 Vejle, Denmark; ^2^Institute of Regional Health Services Research, University of Southern Denmark, 5230 Odense, Denmark; ^3^Department of Obstetrics, Gynecology and Reproductive Sciences, Yale University, School of Medicine, New Haven, CT 06510, USA; ^4^Department of Pathology, Vejle Hospital, 7100 Vejle, Denmark; ^5^Department of Pathology, Yale University School of Medicine, New Haven, CT 06510, USA; ^6^Reproductive Immunology Unit, Department of Obstetrics, Gynecology and Reproductive Sciences, Yale University, School of Medicine, 333 Cedar Street, LSOG 305A, New Haven, CT 06520, USA

## Abstract

Epithelial ovarian cancer stem cells (EOC stem cells) have been associated with recurrence and chemoresistance. CD44 and CK18 are highly expressed in cancer stem cells and function as tools for their identification and characterization. We investigated the association between the number of CD44+ EOC stem cells in ovarian cancer tumors and progression-free survival. EOC stem cells exist as clusters located close to the stroma forming the cancer stem cell “niche”. 17.1% of the samples reveled high number of CD44+ EOC stem cells (>20% positive cells). In addition, the number of CD44+ EOC stem cells was significantly higher in patients with early-stage ovarian cancer (FIGO I/II), and it was associated with shorter progression-free survival (*P* = 0.026). This study suggests that quantification of the number of EOC stem cells in the tumor can be used as a predictor of disease and could be applied for treatment selection in early-stage ovarian cancer.

## 1. Introduction

Epithelial ovarian cancer (EOC) is the fourth leading cause of cancer-related deaths in women in the United States and the leading cause of gynecologic cancer deaths with a 5-year survival of only 30–40% [1–5]. Most patients are diagnosed with advanced-stage disease and the majority recurs despite optimal surgical debulking and initial response to chemotherapy. Recurrence is almost always accompanied by the development of chemoresistance and carcinomatosis, which may not be amenable to surgery [2]. Thus, patients with recurrent ovarian cancer usually succumb to the disease. 

Current studies suggest that the tumor is initiated and maintained by a unique population of cells with stem-like properties [6]. The cancer stem cell (CSC) hypothesis implies that the inherently chemoresistant CSC can persist after chemotherapy and repopulate the tumor leading to recurrence [7–10]. Contrary to the stochastic model of cancer (clonal expansion), the Cancer Stem Cell model holds that tumors are hierarchically organized and only some cells have the capacity to indefinitely self-renew and sustain tumor growth [11, 12]. It is thought that CSCs are able to survive conventional chemotherapies, which usually target fast dividing cells, and give rise to recurrent tumors that are more resistant and more aggressive [13]. Thus, detection of the CSC population has implications for the diagnosis and treatment of most cancers.

One of the major problems in elucidating the cellular origin and pathogenesis of ovarian cancer is that it is a heterogeneous disease. Indeed, ovarian cancer can be classified into multiple types (serous, endometrioid, clear cell, and mucinous), with each type having widely different clinicopathologic properties. It is therefore possible that each of these types of ovarian cancer has different cellular origin. Consequently, the CSC population for each type may also be variable. It is therefore not surprising that stem cell properties have been reported in ovarian cancer cells isolated using different cell surface markers, including CD44, CD133, or CD24 [14–21]. Each of these ovarian cancer cell types may represent either a hierarchy of CSC or an entirely different population of CSC for that particular ovarian histotype.

Recently we, and others, demonstrated the presence of epithelial ovarian cancer stem cells (EOC stem cells) in tissue samples and cell lines [16–19, 22]. Several markers have been used for the identification of EOC stem cells, which reflect the heterogeneity of ovarian cancer. These markers include CD44, CD133, CD24, ALDH1, MyD88, and CD117. Of these markers, the cell surface protein CD44 has been most extensively described to potently enrich the EOC stem cells. CD44+ EOC stem cells express pluripotency markers such as *β*-catenin, Oct-4, and SSEA-4 [14] and have been demonstrated to be the chemoresistant progenitors *in vivo *and are able to differentiate into the heterogenous cell types comprising the tumor [14, 22]. 

The objectives of the present study were twofold: (i) to characterize the location of CD44+ EOC stem cells in tissue samples and (ii) to determine whether the CD44+ EOC stem cell “load” correlates with clinical parameters in ovarian cancer patients. Using ovarian cancer tissue sections from 117 patients with primary disease, we investigated the relationship between the number of CD44+ EOC stem cells and various clinical parameters, which include chemoresponse and progression-free survival.

## 2. Materials and Methods

### 2.1. Ovarian Cancer Cells

The experiments described here were performed using five EOC stem cells (CD44+) and five mature ovarian cancer cells (mEOC cells, CD44−) that our laboratory isolated and established from either ascites or ovarian tumors [14]. mEOC cells correspond to the CD44− component of the tumor or from cells derived from CD44+ EOC cells following *in vitro *and *in vivo* differentiation. We found the same characteristics in CD44− cells isolated from the original tumor or CD44− cells originated from CD44+ EOC cells following *in vitro* and *in vivo* differentiation [15].

We generated fluorescence-labeled EOC stem cell clones by stable transfection with lentiviral constructs expressing the red fluorescence protein Tomato under the ubiquitin promoter-driven L2G (pFU-L2T) as described elsewhere [23]. This construct led to the most efficient or stable labeling and brightest bioluminescent signal [24].

### 2.2. Protein Preparation

Protein extraction was done as previously described [25]. Briefly, cell pellets were lysed on ice in 1× phosphate-buffered saline with 1% NP40, 0.1% SDS and freshly added 20 mL/mL protease inhibitor cocktail (Sigma Chemical, St Louis, MO, USA) and 2 mM phenylmethylsulfonyl fluoride (Sigma Chemical). Protein concentration was determined by BCA Protein Assay (Pierce Biotechnology, Rockford, IL, USA), and proteins were stored at −80°C until further use.

### 2.3. SDS–PAGE and Western Blots

A quantity of 20 *μ*g of each protein sample was denatured in sample buffer and subjected to 12% SDS-polyacrylamide gel electrophoresis (PAGE) as previously described [25]. The following antibody dilutions were used: CD44 antibody (1 : 2000) MEM-263 (Novus Biologicals, Littleton, CO, USA), monoclonal Ck18 antibody DC10 (1 : 1000) (Cell Signaling, Danvers, MA, USA), and rabbit anti-human *β*-actin (1 : 10,000). Specific protein bands were visualized using enhanced chemiluminescence (Pierce Biotechnology).

### 2.4. Flow Cytometry

Flow cytometry analysis was performed as previously described [14]. Briefly, cells were trypsinized and pelleted cells were incubated with either PE-anti CK18 or APC-anti CD44 antibodies (eBioscience, San Diego, CA). Data was acquired using BD FACS Calibur and analyzed using Cell Quest Pro (BD Bioscience).

### 2.5. Study Population

Tumor tissue and patients' clinical data were collected from a prospective translational research protocol. The patients were all newly diagnosed with ovarian cancer and referred for first-line platinum-based chemotherapy at the departments of clinical oncology at Vejle, Aalborg, Odense, and Herning Hospitals. Collected data were entered into case report forms and all tumor specimens underwent central pathology evaluation. Patients received both oral and written study-related information before they signed a consent form prior to collection of biological material. The Danish Biomedical Research Ethics Committee and the Danish Data Protection Agency approved the study. 

A majority of the patients underwent primary debulking surgery, while a minor portion (*N* = 3, 2.6%) were treated with neoadjuvant chemotherapy. All the patients in this cohort received first-line combination chemotherapy with carboplatin (AUC5) and paclitaxel (175 mg/m²). Treatment was administered every 3 weeks for at least four cycles. Response to chemotherapy was assessed according to GCIG CA125 criteria [26, 27] and/or RECIST criteria by CT or MRI scans. 

### 2.6. CD44 and Ck18 Immunohistochemical Staining

Formalin-fixed, paraffin-embedded tissue blocks obtained during primary tumor debulking and prior to first-line chemotherapy were used for immunohistochemical staining for CD44 or Ck18 (Cell Signaling) 1 : 100 dilution. The slides from the primary debulking operations were collected from nine regional Danish Departments of Pathology and underwent central pathology revision (MW). The tumors were classified according to the WHO histological classification and graded according to Shimizu et al. [28]. One representative paraffin-embedded formalin-fixed tumor block from each patient was selected and 4 *μ*m sections were cut and stored at −80°C until further analysis. One section from each patient was used for IHC with monoclonal CD44 antibody (1 : 2000) MEM-263 (Novus Biologicals, Littleton, CO, USA) and monoclonal Ck18 antibody DC10 (1 : 1000) (Cell Signaling, Danvers, MA, USA). 

In brief, the sections were deparaffinized in Tissue clear (Tissue Tec, Sakura Finetek, Zoeterwoude, Netherlands) followed by washes in a graded series of ethanol for rehydration in Tissue Tec Prism (Sakura, Prohosp, Vaerloese, Denmark). The sections were then treated with 3% H_2_O_2_ to block endogenous peroxidase activity. Heat-induced epitope retrieval was done in a microwave oven using TEG pH 9.0 with 15 minutes boiling and 15 minutes for cooling down. The sections were incubated with the primary antibody in 1% bovine serum albumin/tris-buffered saline for 30 minutes at room temperature. Immunohistochemical staining was performed by the Autostainer Plus Link (AS 10030 DAKO, Glostrup, Denmark) according to manufacturer's instruction. DAKO Envision+ (DAKO, Glostrup, Denmark) was used for antibody detection and was followed by visualization with DAB+ (DAKO, Glostrup, Denmark). After washing, the reaction was enhanced by 0.5% copper sulphate in TBS for 10 minutes, and the slides were counterstained with Mayers sour hematoxyline before dehydration and mounting. 

To validate the immunohistochemical procedure, negative and positive controls were included in each run. A small tissue microarray containing ovarian tumors was used together with tissue from appendix and tonsil for positive controls. The same tissue was incubated in 1% bovine serum albumin/tris-buffered saline but without the primary antibody for negative control.

### 2.7. Evaluation of CD44 Immunohistochemical Staining

The study pathologist (MW) scored all the samples: the whole tumor slide was evaluated, and the percentage of CD44 positive stained cells was divided into 0%, >0–5%, >5%–10%, >10%–20%, >20%–50%, and >50%. For classification, we divided the patients into those with <20% CD44+ cells and those with >20% CD44+ cells.

### 2.8. Statistical Analyses

The correlation between CD44 expression and clinicopathological parameters was assessed by *χ* ^2^ statistics and the same applied to the association between CD44 expression and response to chemotherapy. Progression-free survival was defined as the elapsed time from date of diagnosis (date of primary surgery) until progression or death attributable to any cause. Univariate progression-free survival analysis was performed using the Kaplan-Meier estimates and log-rank statistics for comparison of survival plots. Multivariate progression-free survival analysis was determined by the Cox regression model. The parameters entered in the Cox analysis were CD44 status (Low expression: <20% positive cells; high expression: >20% positive cells), FIGO stage, grade, and residual tumor as categorical variables, and age at diagnosis as a continuous variable. Statistical analyses were performed with the NCSS software (version 2007, Kaysville, Utah, http://www.ncss.com/). A value of *P* < 0.05 was considered statistically significant. 

## 3. Results

### 3.1. Characteristics of Ovarian Cancer Stem Cells

**Figure 1:**
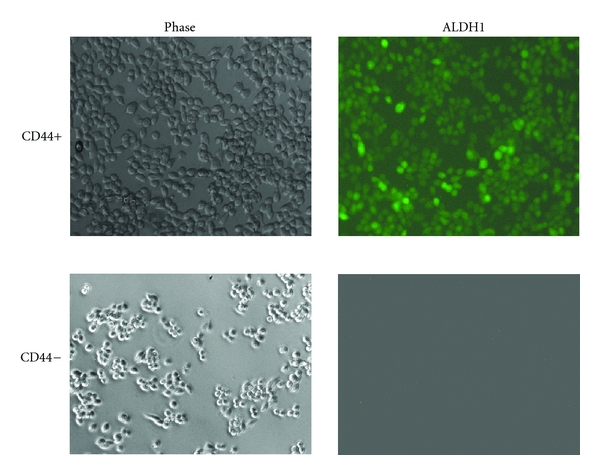
Correlation of CD44 and ALDH1 Expression in epithelial ovarian cancer stem cells. A panel of ovarian cancer cells was evaluated for the expression of CD44 and ALDH1 by immunofluorescence. Only CD44+ EOC stem cells are also positive for ALDH1 expression. representative figure of five independent experiments using five clones of CD44+ cells and their derived CD44− cells.

We previously demonstrated that the ovarian cancer stem cells are CD44+, represent the chemoresistant population, and are able to differentiate *in vitro* and *in vivo* to CD44− cells [14]. Recent studies have shown that ovarian cancer stem cells are also ALDH1+. Therefore, we evaluated ALDH1 expression on CD44+ and CD44− EOC cells. As shown in Figure 1, CD44+, but not CD44−, EOC cells express high levels of ALDH1, further confirming that the CD44+ EOC stem cells express the majority of identified markers for tumor initiating cells (Figure 1) [15, 21].

**Figure 2:**
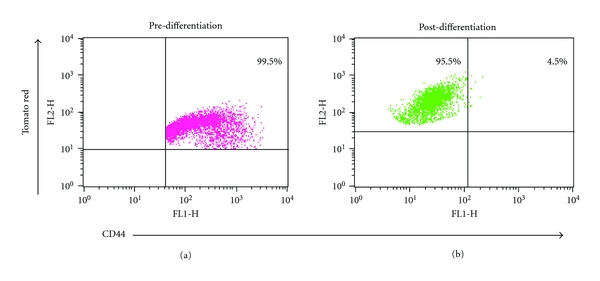
CD44+ EOC cells undergo *in vivo* differentiation into CD44− cells. Flow cytometry analysis of CD44+ cells stable transfected with a lentivirus expressing the fluorescent protein Tomato (red). (a) Cells prior to injection into the mice are 99.5% double positive for CD44 and Tomato. (b) 95.5% of the cells isolated from the tumor remain positive for the fluorescence protein Tomato but are negative to CD44. Only 4.5% of the injected cells remained double positive.

To closely monitor the process of differentiation, we labeled pure clones of CD44+ EOC cells with a fluorescent reporter, which allows flow cytometry analysis and *in vivo* imaging. Thus, CD44+ EOC stem cell clones were stably transfected with a viral vector expressing the red fluorescence protein “Tomato” (pFU-L2T) [23]. CD44+/Tomato+ EOC cells were injected into nude mice, and the established tumor (60 days later) was evaluated for CD44 and Tomato. As shown in Figure 2, prior to injection the EOC stem cells are 99.5% CD44+/Tomato+. The xenograft established, however, is only 4.5 % CD44+/Tomato+ and 95.5% CD44−/Tomato+. These results demonstrate that the CD44− cells originated from the CD44+/Tomato+ EOC cells (Figure 2).

### 3.2. Cytokeratin 18 (Ck18) Is Preferentially Expressed by the EOC Stem Cells

**Figure 3:**
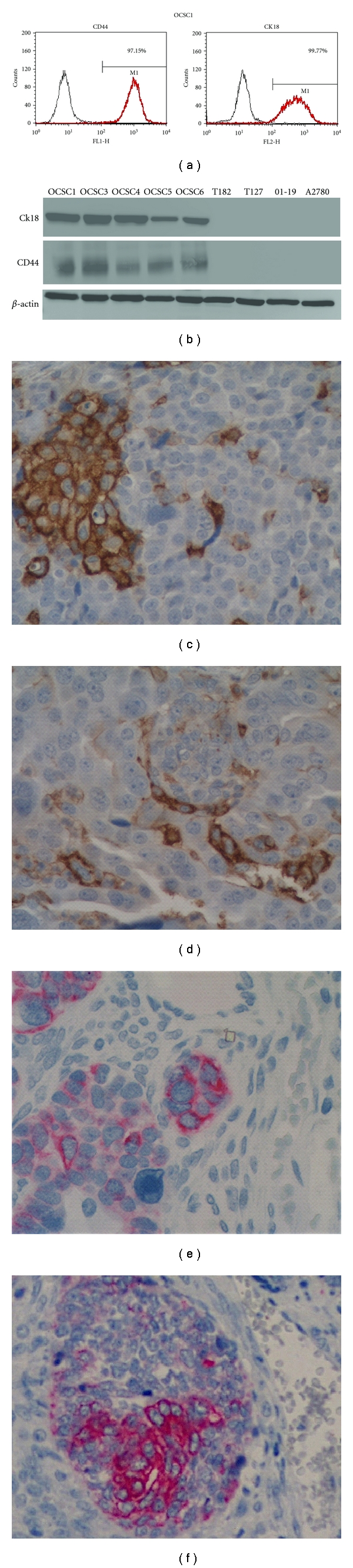
Expression of CD44 and CK18 in multiple clones of ovarian cancer cells. (a)-(b). CD44+ cells also express CK18 as determined by flow cytometry (a) and western blot analysis (b). Flow cytometer is representative of the eight evaluated clones. EOC stem cells, determined by either CD44 (c and d) or Ck18 (e and f) expression, are found in clusters surrounded by CD44− or Ck18− negative cancer cells.

**Figure 4:**
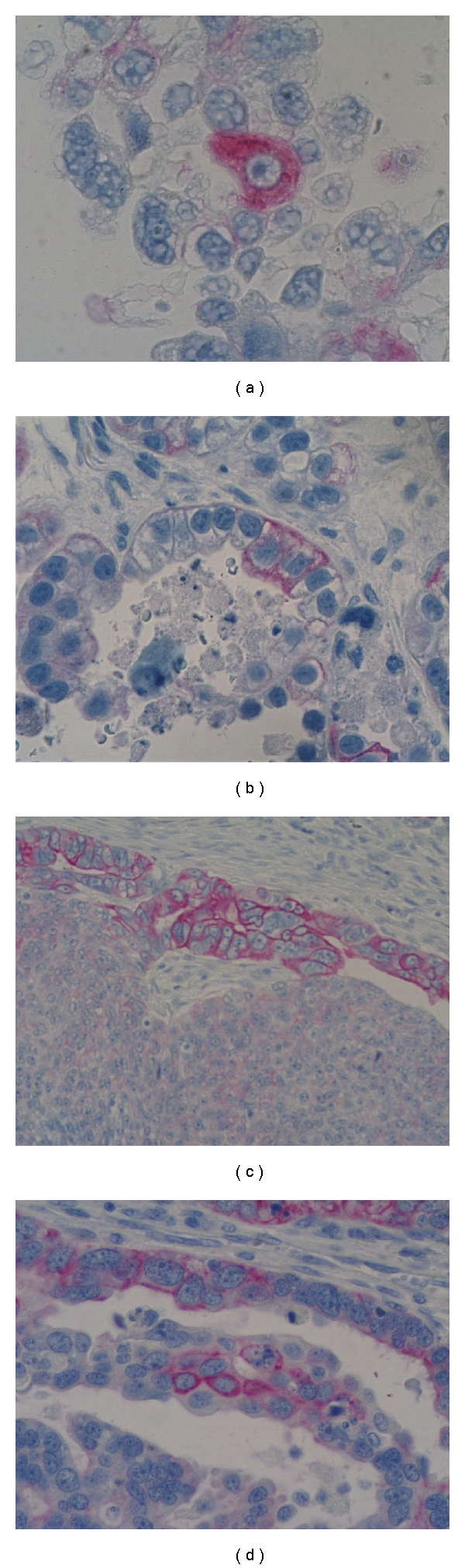
EOC stem cells present unique morphological characteristics. CD44+ EOC stem cells are characterized by high nuclear to cytoplasm (N/C) ratio, contain vesicular chromatin pattern, and have prominent nucleoli. The cells can be found as single cells (a) or clusters of two or more cells (b–d).

**Figure 5:**
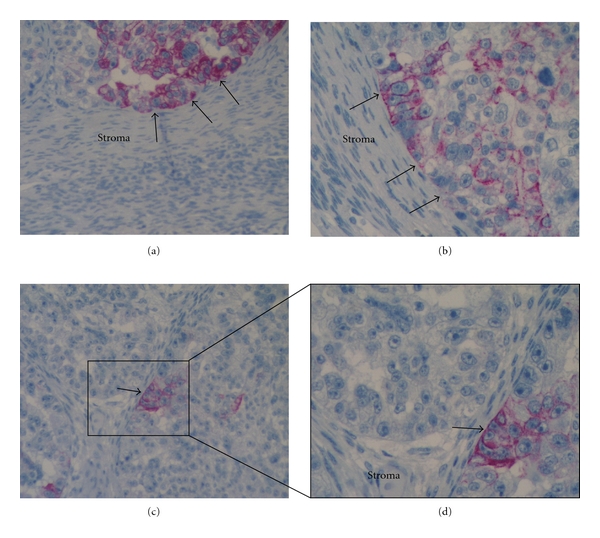
Association of EOC stem cells with stroma. (a-b) Ck18+ EOC stem cells are in close contact with the surrounding stroma (arrows); (c) a cluster of Ck18+ EOC stem cells are in close contact with the stroma forming a niche; (d) magnification of (c) showing the different cellular components: EOC stem cells (in red) in direct contact with the stroma and surrounded by Ck18-negative cancer cells. The arrow shows the basement membrane between the two compartments.

We previously showed, using gene expression microarray, that Ck18 expression is 7-fold higher (*P* = 0.0007) in CD44+/MyD88+ EOC stem cells compared to the CD44−/MyD88− mature ovarian cancer stem cells (mOCCs) [14]. To validate this finding, we determined the levels of Ck18 in five EOC stem cell clones, three mOCC clones, and in the EOC cell line A2780 using western blot analysis. As shown in Figure 3, Ck18 expression is limited to the EOC stem cells and not the mOCCs. Correlation between CD44 and Ck18 expression was also observed by flow cytometry and western blot (Figures 3(a), 3(b)). Evaluation of the location of CD44+ and Ck18+ cells in tumor tissues obtained from ovarian cancer patients showed that CD44+ (Figures 3(c), 3(d)) and Ck18+ cells (Figures 3(e), 3(f)) are surrounded by CD44-/Ck18− mOCCS. Within tumor nests, single (Figure 4(a)) and clusters (Figures 4(b)–4(d)) of Ck18+ cancer cells were observed. These cells morphologically appear less differentiated with larger size, higher nuclear to cytoplasm (N/C) ratio, more prominent nucleoli, and a vesicular chromatin pattern (Figure 5). In cells with a more differentiated phenotype (smaller size and lower N/C ratio), Ck18 staining was weak to absent (Figures 3 and 4). Some of the Ck18+ clusters were observed in close proximity to the stroma and showed a clear and defined basal membrane (Figure 5). The observed distribution of the Ck18+ cancer cells follows the description of the niche associated with CSC [9, 29]. A similar pattern of localization was observed with CD44 staining [14]. 

### 3.3. Variable Expression of CD44+ EOC Stem Cells in Ovarian Cancer Tissues 

Our next objective was to determine whether the prevalence of EOC stem cells has a prognostic value. For this study, we focused using a single marker and selected CD44 as a widely accepted marker for the identification of ovarian cancer stem cells. Thus, we analyzed CD44 staining in ovarian cancer tissue sections obtained from 117 patients. The clinical-pathological data of the study cohort is presented in Table 1. The majority of the patients were older than 50 years with histopathologic diagnosis of serous ovarian cancer. In addition, most of the patients were classified FIGO stage II and higher, with moderate or poorly differentiated tumors (grade > 1) (Table 1). We detected CD44+ cancer cells in all but one tissue section tested. However, we observed variability in the number, distribution, and location of CD44+ cancer cells amongst patients. Of all patients tested, 39 patients had between 1–5% CD44+ cancer cells, 38 patients had >5–10% CD44+ cells, 19 patients had >10–20% CD44+ cells, 9 patients had >20–50% CD44+ cells, and 11 patients had >50% CD44+ cells. Only one sample was negative for CD44 staining. Due to this high variation, we classified the samples as low expression of EOC stem cells if they had less than 20% positive cells (<20% EOC stem cells) and high expression if the sample had more than 20% positive cells (>20% EOC stem cells). Thus, of the 117 patients, 20 patients were considered high expression and 97 patients were considered low expression (Table 1). We then determined if the percentage of EOC stem cells has clinical correlation.

### 3.4. CD44 Levels Inversely Correlate with FIGO Stage and Tumor Grade 

Patients with FIGO stage I tumors had a higher number of CD44+ EOC stem cells (>20% CD44+ cells) with 57.1% of the stage I patients expressing >20% CD44+ cells. For FIGO stages II, III, and IV, 18.2%, 12.9%, and 4.5% expressed >20% CD44+ cells (Table 1). Thus, a significant percent had FIGO stage I (*P* = 0.00025; *x* ^2^ = 19.2). Similarly, the majority of patients with grade I tumors showed high expression of EOC stem cells (>20% CD44+ cells) (*P* = 0.021, *x* ^2^ = 7.7, Table 1). High expression of CD44+ EOC cells was seen in fifty percent, 14.3%, and 12.1% of grade 1, 2, and 3 disease, respectively. This indicates that in patients with primary disease, tumors tend to have a lower number of CD44+ EOC stem cells as the disease progresses. 

### 3.5. Correlation between Number of CD44+ EOC Stem Cells and Chemoresponse

We then evaluated whether a correlation exists between percentage of CD44+ EOC stem cells and response to treatment. All the patients in this cohort received treatment. The 14 patients with FIGO stage I cancer comprised 2 patients with stage IA cancer (one clear cell cancer and one grade 2 serous = patients with adverse histological features/high risk patients that routinely receive chemotherapy) and 12 patients with stage IC tumors that according to guidelines are treated with adjuvant chemotherapy. Although it was only marginally statistically significant, we observed an obvious trend (*P* = 0.06) for poorer response rates among patients with >20% CD44+ EOC stem cells. Only 73% of these patients had complete or partial response compared to 90% in patients with low number of EOC stem cells (<20% CD44 positive cells). Similarly, 27% of patients with >20% positive cells for CD44 had stable or progressive disease during or by the end of first line carboplatin and paclitaxel treatment compared to only 10% in the patients with a low number of CD44+ cells (<20% CD44+ EOC stem cells) (Table 2).

### 3.6. Correlation between the Number of CD44+ EOC Stem Cells and Progression-Free Survival

Although a majority of patients with early-stage ovarian cancer respond to treatment and have a good prognosis, 10% of these patients will recur in spite of appropriate debulking and chemotherapy. Thus, in order to determine whether there is a correlation between the presence of EOC stem cells and recurrence, we analyzed our study population with respect to progression-free survival. In multivariate analysis, the percentage of CD44+ EOC stem cells was independently correlated to progression-free survival with a hazard ratio of 2.44 (1.08–5.52) 95% CI, toward shorter survival for patients with high number of EOC stem cells (Table 3). As anticipated, FIGO stage and residual tumor were also independently correlated to progression-free survival. 

**Figure 6:**
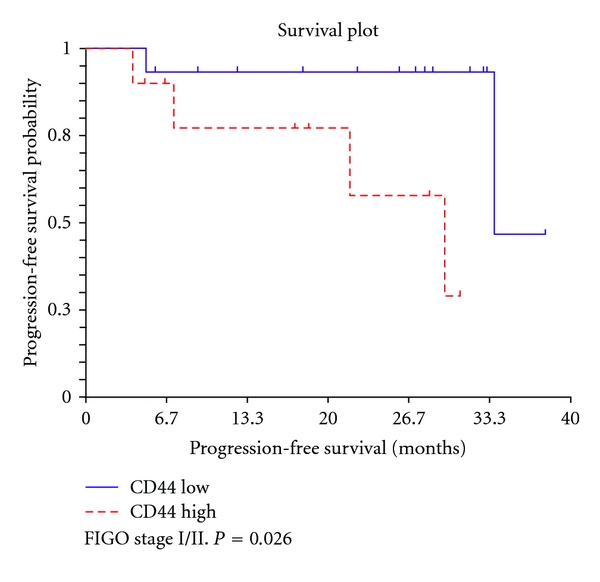
Progression-free survival in ovarian cancer patients with stage I/II. In early-stage ovarian cancer (FIGO stage I/II), patients with a high percentage of CD44+ positive EOC stem cells (>20%) (CD44 High) had significantly shorter progression-free survival compared to patients with a low number of CD44+ EOC stem cells (CD 44 low) (*P* = 0.026).

Further subgroup analysis showed that in early-stage ovarian cancer (FIGO stage I/II), patients with high number of CD44+ EOC stem cells (>20%) had significantly shorter progression-free survival compared to patients with a low number of CD44+ cells EOC stem cells (<20%) (*P* = 0.026) (Figure 6). In contrast, in patients with advanced-stage ovarian cancer (FIGO stage III/IV), the number of CD44+ EOC stem cells did not correlate with progression-free survival (*P* = 0.95, data not shown).

## 4. Discussion

We show in this paper that CD44+ EOC stem cells can be detected in tumor sections obtained from patients with ovarian cancer. Interestingly, in this retrospective study, we found that there was an inverse correlation between FIGO stage/disease grade and the presence of these cells. However, our findings show that in early-stage ovarian cancer, patients with tumors containing >20% CD44+ EOC stem cells had a shorter progression-free survival compared to patients with tumors having <20% of these cells. In multivariate analysis, we found that CD44 positivity was an independent predictor of poor progression-free survival. Since high levels of CD44+ EOC stem cells correlated with poor prognosis in early stage ovarian cancer but not in patients with advanced FIGO stage, it is possible that the high level of EOC stem cells in stage I, and II resulted in the observed significance in the multivariate analysis.

The existence of CSC has been demonstrated in several tumor types such as acute myelogenous leukemia, breast, pancreatic, and brain tumors [11, 30–33]. These cells are believed to sustain tumor formation through their self-renewal and differentiation potential. In ovarian cancer, Bapat et al. [16] reported the isolation and identification of ovarian CSC. Using an *in vitro *model system comprised of 19 spontaneously immortalized clones derived from an advanced-grade patient, the authors demonstrated the ability of 2 clones to form spheroids and recapitulate the human tumor in nude mice. These cells were shown to express CD44, E-cadherin, and the stem cell factors Nestin, Nanong, and Oct-4. In a separate study, Zhang et al. [18] reported the tumor-initiating capacity of CD44+/CD117+ ovarian cancer cells in mice. 

The identification of CSC is done based on the presence of extracellular markers that are thought to be stem cell specific. Some of the most commonly identified markers are CD133, CD44, and CD24, which are found in breast, prostate, pancreas, and ovarian cancer. Although these markers are thought to be indicative of CSC phenotype, it is not clear whether they are universal markers and if it is a characteristic of CSC derived from all type of tumors. That is the case for ovarian cancer where multiple markers have been described for the isolated tumor initiating cells. A potential explanation for the discrepancy could be due to studies using cancer cells lines which may not represent the original tumor. However, it may also be a result of the heterogeneous nature of ovarian cancer and its multiple sources of origin [34, 35]. An ovarian cancer stem cell originated in the fallopian tube might present different surface markers than a CSC originated from the endometrium or the surface epithelium of the ovaries. 

Our group previously identified at least two types of EOC cells based on their response to chemotherapy: Type I-chemoresistant and Type II-chemosensitive EOC cells [36, 37]. Further characterization showed that these cells have additional differences in terms of growth, cytokine production, and intracellular markers [38]. While Type II EOC cells represent the “classical” ovarian cancer cells characterized by fast growth and lack of cell to cell contact inhibition, Type I EOC cells are characterized by slower growth, which is inhibited upon cell to cell contact. In addition, Type I, but not Type II, EOC cells have constitutive NF-kB activity and constitutively secrete IL6, IL8, MCP-1, and GRO*α* [14]. Gene expression microarray analysis comparing these two types of cells further showed that Type I EOC cells expressed significantly higher levels of the stem cell markers, CD44 and SSEA-4, the TLR adapter protein MyD88, Cytokeratin 18, Trop-1, and others [14]. In contrast, Type II EOC cells were negative for all these markers.

These findings suggest that Type I EOC cells may represent the population that has stem-like properties. Indeed, we demonstrated that Type I EOC cells, as selected by CD44, are able to form xenografts in mice and resulted in tumors containing both CD44+ and CD44− cells. In this study, we evaluated additional markers present in our recently isolated CD44+ EOC cells [14]. We observed that these cells are also CK18+, a marker associated with epithelium of the fallopian tubes. 

Aldehyde dehydrogenase (ALDH1) has been proven useful for the identification of cancer stem cells, including ovarian cancer [19]; therefore, we evaluated the expression of ALDH1 in the identified CD44+ EOC stem cell clones. We found high levels of ALDH on EOC stem cells by immunofluorescence, suggesting that ALDH1 could be used also as a marker to monitor the presence of cancer stem cells. 

We described additional evidence in support of previous findings showing that Type I EOC cells (CD44+) are the source of Type II cells or CD44−. To closely monitor EOC stem cell fate and function in mice, we labeled CD44+ cells with dual-function reporter genes encoding the sequence of the florescence protein Tomato (red color). Using a xenograft tumor model, we demonstrated that following injection of double positive CD44+/Tomato+ cells, the newly formed tumor originating from these double positive cells is characterized by CD44− cells, which maintain the expression of the fluorescent protein Tomato. This demonstrates that CD44+ EOC stem cells can both self-renew and differentiate [39]. Moreover, microscopic analysis of the xenografts showed that CD44+ EOC cells were able to recapitulate the morphology of the original tumor [14]. Finally, *in vitro* differentiation of the chemoresistant CD44+ EOC stem cells resulted in chemosensitive cultures that have lost CD44. In this study, we showed that the presence of CD44+ EOC stem cells correlates with poor prognosis. Since the CD44+ cells are in general more chemoresistant, they can persist after chemotherapy and may initiate recurrence upon the completion of treatment. 

We found EOC stem cells localized in clusters surrounded by differentiated ovarian cancer cells and in close proximity with the stroma. Emerging evidence indicates that a specialized environment, the stem cell niche, is one of the factors regulating stem cell maintenance and self-renewal [9, 40, 41]. Alterations to the stroma may affect the control of self-renewal [30]. This is illustrated by the studies of Yauch et al. who showed that inhibition of Hh pathway in pancreatic associated stroma cells resulted in suppression of tumor growth [42]. In contrast, inhibition of the same pathway in the cancer cells did not have any effect on tumor growth. This suggests that the variation on the number of cancer stem cells observed in our study may be the result of alteration in the interaction between the stroma and the cancer stem cells. A functional stroma might maintain a small pool of cancer stem cells while promoting differentiation. However, disruption of the stroma-cancer stem cells interaction might lead to uncontrolled self-renewal and significant increase in the pool of chemoresistant EOC stem cells and consequent poor prognosis. 

CD44 is a cell surface glycoprotein receptor with several isoforms [43]. All isoforms are encoded by a single gene and result from alternative splicing. CD44 is expressed by most cells, including hematopoietic cells and tumors. Several studies have evaluated CD44 expression in ovarian cancer tumors and correlated with survival outcome. CD44 expression has been reported to correlate with a significantly shorter disease survival than for patients with CD44 negative tumors [44, 45]. However, studies investigating CD44 expression in terms of IHC and survival are contradictory [46, 47]. Differences between these studies that could account for differences in their findings could be attributed to technical factors, including the use of different monoclonal or polyclonal antibodies that exhibit variable efficacy in paraffin-embedded tissues and to different methods used for assessment of immunostaining. In this study, we focused on CD44 expression as a marker of the cancer stem cells and its evaluation is based on the percentage of ovarian cancer stem cells present in the tumor. 

CD44 is more than a marker; this transmembrane receptor has been shown to be important in various cellular processes such as growth, differentiation, and motility [43]. The most studied function of CD44 is its role as the receptor for hyaluronan (HA) [48]. Binding of HA to CD44 controls cell-cell interactions, as well as interactions of the cell with the extra-cellular matrix. Furthermore, it can function as detector of tissue damage and promote tissue repair. Therefore, it is possible that CD44 expression in EOC stem cells might play a central role in self-renewal and the response to tissue damage.

## 5. Conclusion

We describe the intratumoral localization of EOC stem cells in ovarian tumor samples. We show their existence as clusters located close to the stroma forming what has been described as the CSC “niche”. Furthermore, we demonstrate a correlation between the percentage of CD44+ EOC stem cells and survival in early-stage ovarian cancer. Although it is a small cohort, especially the early stage, the findings from this study are important since they suggest that quantification of the number of EOC stem cells present in the tumor can be used as a predictor of disease and could be applied for treatment selection in early-stage ovarian cancer.

##  Conflict of Interests 

The authors declare that there is no conflict of interests.

## Figures and Tables

**Table 1:** Patient and tumor characteristics and percentage of CD44+ EOC stem cells. Groups are classified based on their CD44 expression as tumors containing less than 20% CD44 positive (>20%) or more than 20% CD44 positive cells (<20%).

Characteristics	No. of patients	%	<20% CD44 Cells N (%)	>20% CD44 Cells N (%)	*P*
Age					0.0013
<50	10	8.6	6 (60.0)	4 (40.9)	
51–65	54	46.2	40 (74.1)	14 (25.9)	
>65	53	45.3	51 (96.2)	2 (3.8)	
Median 63.2					
Range 32–79					
FIGO stage					0.00025
I	14	12.0	6 (42.9)	8 (57.1)	
II	11	9.4	9 (81.8)	2 (18.2)	
III	70	59.8	61 (87.1)	9 (12.9)	
IV	22	18.8	21 (95.5)	1 (4.5)	
Tumor grade					0.0209
1	8	6.8	4 (50.0)	4 (50.0)	
2	42	35.9	36 (85.7)	6 (14.3)	
3	58	49.6	51 (87.9)	7 (12.1)	
Not graded (clear cell or metastatic biopsy)	9	7.7			
Histopathologic cell type					0.0760
Serous	100	85.5	86 (86.0)	14 (14.0)	
Endometrioid	5	4.3	4 (80.0)	1 (20.0)	
Clear cell	8	6.8	5 (62.5)	3 (37.5)	
Mucinous	1	0.9	0 (0.0)	1 (100)	
Carcinomas (mixed or undifferentiated)	3	2.6	2 (66.7)	1 (33.3)	
Residual postoperative tumor					0.014
≤1 cm	56	51.4	42 (75.0)	14 (25.0)	
>1 cm (Unknown: 8 )	53	48.6	49 (92.5)	4 (7.5)	
CD44 immunostaining					
Less than 20% CD44+ cells	97	82.9	NA	NA	NA
More than 20% CD44+ cells	20	17.1			

**Table 2:** Correlation between percentage of CD44+ EOC stem cells and response to first-line carboplatin/paclitaxel treatment. Groups are classified based on their CD44 expression as tumors containing less than 20% CD44 positive (>20%) or more than 20% CD44 positive cells (<20%).

	Percentage of CD44+ EOC stem cells		
	<20% CD44 Cells (*n* = 84)	>20% CD44 Cells (*n* = 15)	*P*
			0.06
CR + PR (*n* = 87)	76 (90%)	11 (73%)	
SD + PD (*n* = 12)	8 (10% )	4 (27%)	

**Table 3:** Multivariate analyses of progression-free survival in 117 ovarian cancer patients.

Clinicopathological characteristics	B regression coefficient	Standard Error (B)	Hazard ratio	95% CI (Hazard ratio)	*P* value*
Age	−0.025	0.014	0.98	0.95–1.00	0.083
FIGO stage					
I/II			1.00		
III/IV	1.814	0.502	6.13	2.29–16.4	0.0003
Grade					
1			1.00		
2	0.750	0.778	2.12	0.46–9.73	0.34
3	0.617	0.769	1.85	0.41–8.36	0.42
Residual tumor					
≤1 cm			1.00		
> cm	0.858	0.277	2.35	0.83–2.30	0.002
% E O C stem cells					
<20% CD44 Cells			1.00		
>20% CD44 Cells	0.893	0.416	2.44	1.08–5.52	0.032

*Cox regression model.
